# Glial Cells in Glaucoma: Friends, Foes, and Potential Therapeutic Targets

**DOI:** 10.3389/fneur.2021.624983

**Published:** 2021-03-16

**Authors:** Mariana Y. García-Bermúdez, Kristine K. Freude, Zaynab A. Mouhammad, Peter van Wijngaarden, Keith K. Martin, Miriam Kolko

**Affiliations:** ^1^Department of Drug Design and Pharmacology, University of Copenhagen, Copenhagen, Denmark; ^2^Department for Veterinary and Animal Science, University of Copenhagen, Copenhagen, Denmark; ^3^Center for Eye Research Australia, Royal Victorian Eye and Ear Hospital, East Melbourne, VIC, Australia; ^4^Ophthalmology, Department of Surgery, University of Melbourne, Melbourne, VIC, Australia; ^5^Department of Ophthalmology, Copenhagen University Hospital, Rigshospitalet-Glostrup, Glostrup, Denmark

**Keywords:** Glaucoma, glia, retinal ganglion cells, Müller glial cells, microglia, astrocytes, oligodendrocytes, retinal glia interactions

## Abstract

Glaucoma is the second leading cause of blindness worldwide, affecting ~80 million people by 2020 (1, 2). The condition is characterized by a progressive loss of retinal ganglion cells (RGCs) and their axons accompanied by visual field loss. The underlying pathophysiology of glaucoma remains elusive. Glaucoma is recognized as a multifactorial disease, and lowering intraocular pressure (IOP) is the only treatment that has been shown to slow the progression of the condition. However, a significant number of glaucoma patients continue to go blind despite intraocular pressure-lowering treatment (2). Thus, the need for alternative treatment strategies is indisputable. Accumulating evidence suggests that glial cells play a significant role in supporting RGC function and that glial dysfunction may contribute to optic nerve disease. Here, we review recent advances in understanding the role of glial cells in the pathophysiology of glaucoma. A particular focus is on the dynamic and essential interactions between glial cells and RGCs and potential therapeutic approaches to glaucoma by targeting glial cells.

## Introduction

Glaucoma is a group of eye diseases that can cause vision loss and blindness. The number of people with glaucoma is increasing due to the age-related nature of the disease (3). Hence, it is estimated that more than 120 million people worldwide will suffer from glaucoma in 2040 (4). Glaucoma is characterized by progressive degeneration of retinal ganglion cells (RGCs) and is often asymptomatic until its advanced stages when vision loss is irreversible (5). Glaucoma can be classified as either primary or secondary, with secondary glaucoma attributable to known pathologies or medications. Glaucoma may be further classified as either open-angle or angle-closure according to the anatomy of the aqueous outflow pathway (2, 6). In all subtypes of glaucoma, the inner retinal degeneration, especially the gradual loss of RGCs, is a hallmark (7). RGCs are the output neurons of the retina, and their axons transfer visual information from the retina to the brain (8). RGC dysfunction and death lead to vision impairment and ultimately to blindness. To date, no approved treatments for glaucoma directly target RGCs. Instead, the only available treatments are indirectly protective for RGCs by lowering the intraocular pressure (IOP). It is, therefore, crucial to identify cellular mechanisms for the prevention of RGC degeneration, the repair of dysfunctional cells, and the promotion of axonal regeneration to limit the projected burden of vision impairment and blindness from glaucoma (9, 10). Although the most investigated risk factors for glaucoma progression include IOP, age, genetic background, thinner corneal thickness, and vascular dysregulation (11), other disease mechanisms such as oxidative stress, mitochondrial dysfunction, excitotoxicity, and immunological processes may contribute to the pathophysiology of the disease (2, 12, 13). In this context, accumulating evidence suggests that glial cells in the retina and optic nerve may play important roles in the pathogenesis of RGCs (14–16). However, despite having been studied for more than a century, there are substantial aspects of the interrelationship between glial cells and RGCs that are still to be elucidated (17, 18).

Emerging literature emphasizes the roles of glia in both the maintenance of the retina and in the pathogenesis of glaucoma (15, 19). Although Müller glia, astrocytes, oligodendrocytes, and microglia have different developmental origins, they are now known to share many functions. Although some functions are subserved simultaneously by different glia, others are performed by specific glial subtypes (14, 19–22). Similarly, attention has turned to the complex interactions between retinal glia and neurons and the centrality of these interactions to retinal homeostasis (14, 19, 23, 24). Likewise, it is evident that the glial response to injury stimuli can further perpetuate RGC damage (17, 23, 25, 26). Despite these important advances in our understanding of the interactions between glia and retinal neurons in health and in the context of glaucoma, there is still much to be learned.

## Glial Cells of the Retina are Not Just Support Cells

Glial cells are named after the Greek word for glue, as it was thought that their function was simply to bind the neurons in the central nervous system (CNS) together (27). It is now understood that glial cells play a range of diverse and complex functions beyond the provision of structural support to neurons. Two basic types of glial cells are found in the human retina: macroglia and microglia. Retinal macroglia are comprised of Müller glia and astrocytes. Macroglia maintain retinal homeostasis by regulating ion exchange, glucose, and neurotransmitter transport (14). Microglia respond to retinal injury and are important in the maintenance of neuronal networks and the mediation of neuroinflammation (14, 28–30). In the optic nerve, oligodendrocytes, another type of macroglia, and astrocytes provide essential support to RGC axons as they travel to the brain (31). Accumulating evidence suggests that both types of glial cells are interacting with the retinal and optic nerves, and are important contributors to the pathophysiological processes leading to glaucomatous RGC loss (14, 15, 17, 23, 32–34).

## A Partnership Between Müller Glia and Retinal Ganglion Cells

**Figure 1:**
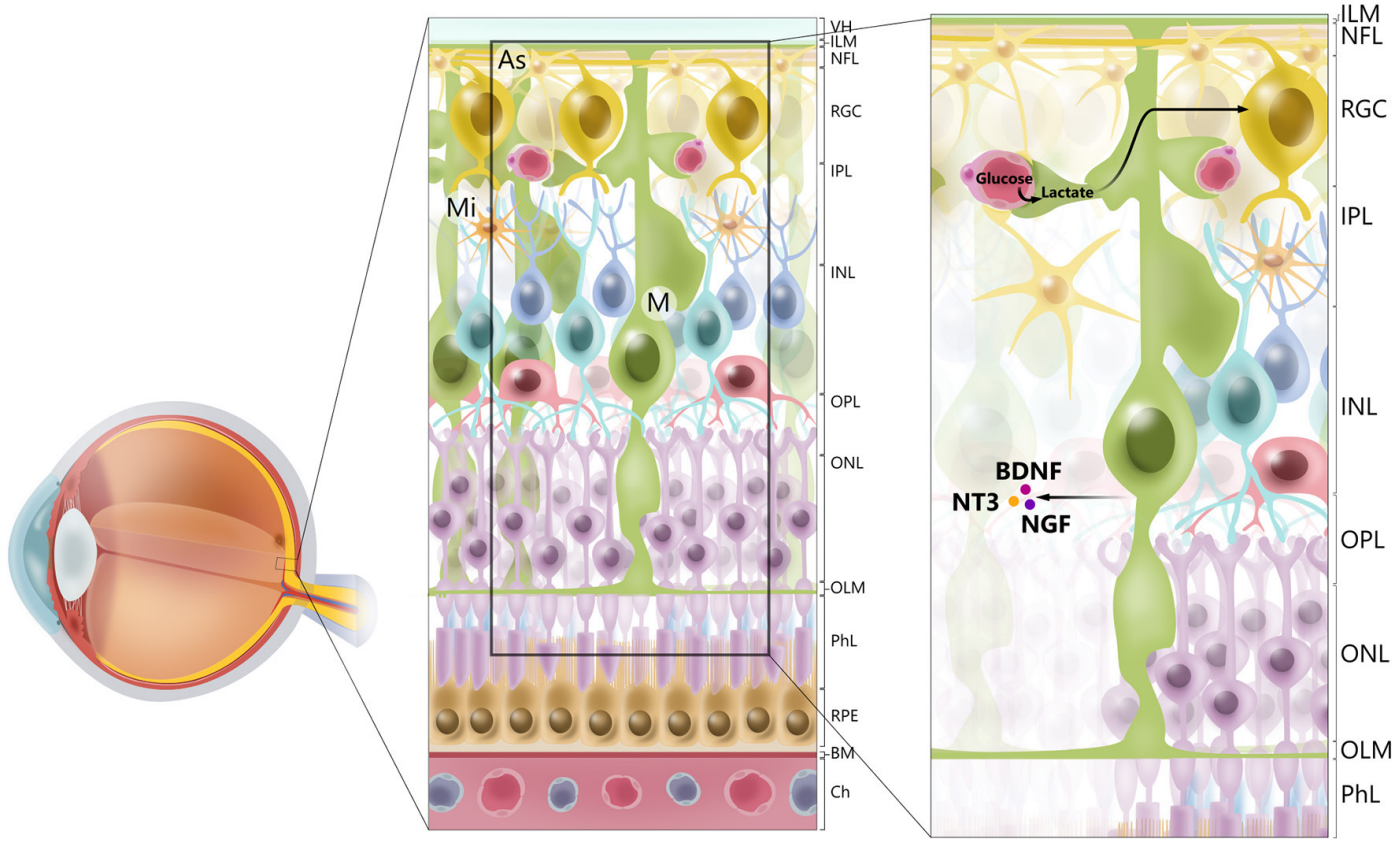
Cellular architecture of mammalian retina. Retina consists of 10 layers. Starting with layer furthest away from vitreous humor (VH), layers of retina are *retinal* pigment epithelium (RPE), photoreceptor layer (PhL), outer limiting membrane (OLM), outer nuclear layer (ONL), outer plexiform layer (OPL), inner nuclear layer (INL), inner plexiform layer (IPL), retinal ganglion cell layer (RGC), retinal nerve fiber layer (RGC axons) (NFL), and inner limiting membrane (ILM). Main types of glial cells found in mammalian retina are macroglia [Müller glia (MG) and astrocytes (As), and microglia (Mi)]. Glial cells serve to maintain retinal homeostasis. As illustrated, Müller glia traverses retina, providing structural support to neurons, mediating transport of molecules between retinal vessels and RGCs, and secreting neurotrophic factors, such as BDNF, NT3, and NGF. Furthermore, processes of Müller glia comprise outer and inner limiting membranes of retina. Müller glia, end-feet of astrocytes and vascular endothelial cells, and basal membrane constitute blood–retinal barrier. BM. basal membrane; Ch, choriocapilaris.

Müller glia are found throughout the retina, with processes extending from the outer limiting membrane to the inner limiting membrane and surrounding the RGCs. Their unique morphology and distribution are related to the role of Müller glia as mediators of the transport of molecules between RGCs and the vitreous humor, retinal vessels, and the subretinal space (15) (Figure 1). Müller glia have multiple functions and are symbiotically associated with RGCs.

### Glutamate Clearance to Avoid Neurotoxicity

**Figure 2:**
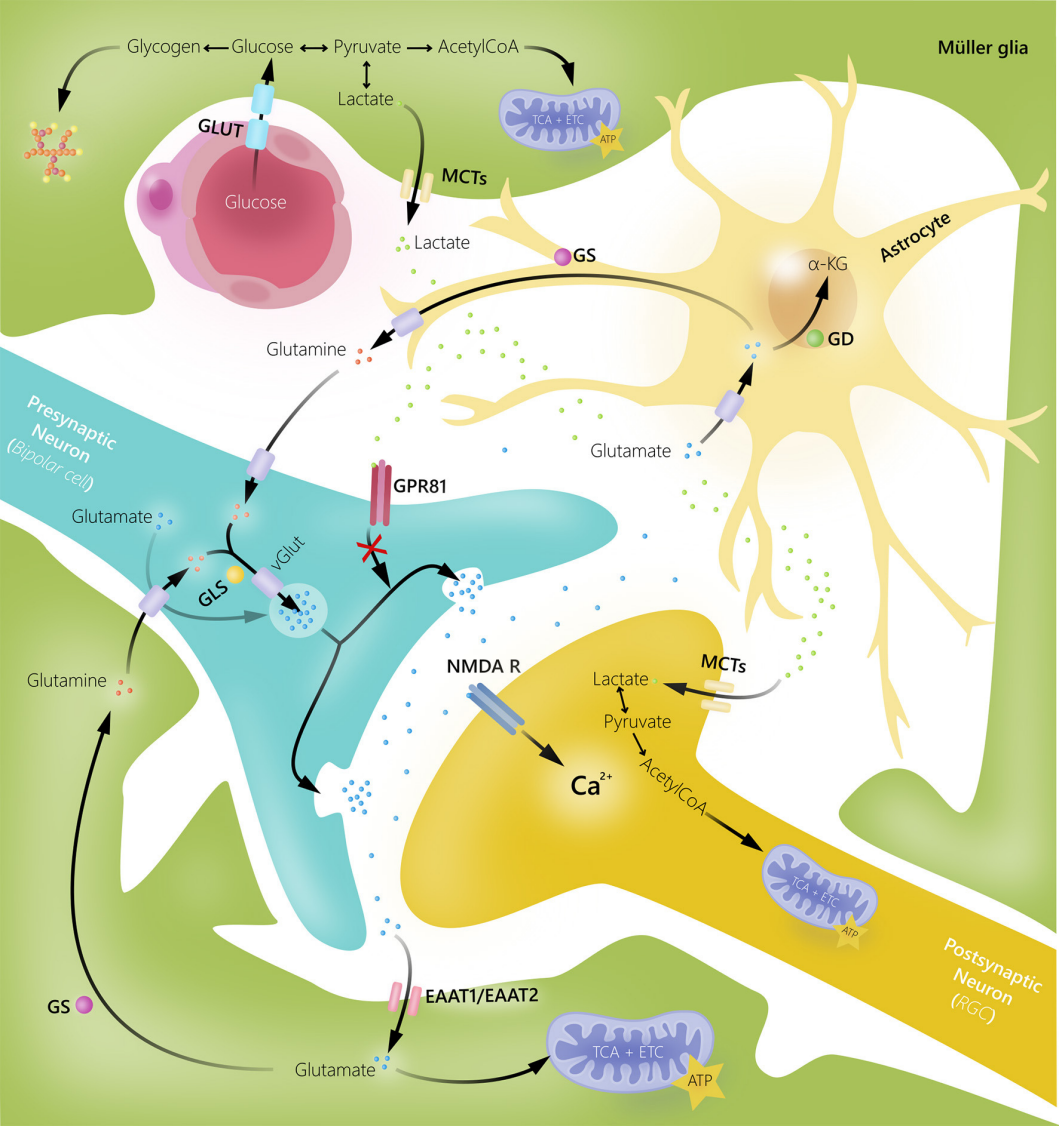
Glial cell and neuronal interactions in human retina. Müller glia and astrocytes take up excess extracellular glutamate to prevent glutamate-induced excitotoxic damage of retinal ganglion cells (RGC). Once glutamate is transported into glial cells, it is converted into glutamine by glutamine synthetase (GS). Glutamine can then be released by glial cells, taken up by neurons, converted into glutamate by glutaminase (GLS), and reused in synaptic neurotransmission. Glutamate can also be converted into α-ketoglutarate by glutamate dehydrogenase (GD) and used as an energy substrate. Müller glia supply bipolar cells and RGCs with energy substrates in form of lactate. Additionally, lactate released by Müller glia may function as a signaling molecule for G-protein coupled receptor 81 (GPR81) to inhibit glutamate release. EAAT, excitatory amino acid transporter; GLUT, glucose transporter; MCTs, monocarboxylate transporters; NMDAR, *N*-methyl-d-aspartate receptor.

An essential role of Müller glia is their ability to rapidly remove excess glutamate from the extracellular space by amino acid transporters (excitatory amino acid transporters), keeping it at low concentrations to avoid excitotoxicity (18, 24, 35, 36). Glutamate is converted to glutamine *via* the glial-specific enzyme glutamine synthetase. Glutamine subsequently serves as the precursor for glutamate in neurons. In patients with glaucoma and some animal models of the disease, an augmentation of glutamine expression in Müller glia has been shown, indicating an enhanced activation of the glutamate–glutamine cycle (37–39). In addition, it is thought that increased glutamine levels in Müller glia might be due to reduced glutamine requirement in damaged RGCs (37). In addition to removing excess glutamate from the synapse, Müller glia can also use glutamate as a metabolic substrate (16, 18, 26) (Figure 2).

### Glial Cell Line-Derived Neurotrophic Factor in Glutamate Homeostasis

Another crucial Müller glia feature is the ability to release neurotrophic factors. In this context, studies have shown that ischemia-induced glial cell activation results in the release of glial cell line-derived neurotrophic factor (GDNF), which increases glutamate uptake, thereby potentially facilitating neuroprotection by reducing glutamate-induced excitotoxicity (37, 40). The potential neuroprotective role of GDNF has been supported in a rat ocular hypertension model, where an intravitreal injection of GDNF-containing microspheres was shown to increase RGC survival while reducing glia cell activation (41). The neuroprotective effect of GDNF has furthermore been associated with reduced activation of the L-glutamate receptor, *N*-methyl-d-aspartate receptor (NMDAR) (42), by receptor desensitization and downregulation in both neocortical neurons and astrocytes through activation of mitogen-activated protein kinase (MAPK) (43, 44). However, there are conflicting results regarding the effect of GNDF on glutamate homeostasis, with one study suggesting that GDNF pre-treatment can increase neuronal cell death *via* upregulation of glutamate transporters with a consequent increased excitotoxic concentration of glutamate (45).

### *N*-Methyl-D-Aspartate Receptor Activation Is Crucial in Retinal Homeostasis

In general, safeguards against glutamate excitotoxicity have been proposed to be important treatment targets to prevent retinal neurodegeneration. In particular, NMDAR activation has been extensively studied and found to be essential for retinal homeostasis but, at the same time, to cause neurodegeneration when overactivated (46). To activate NMDARs, D-serine, or glycine along with glutamate are required. D-serine is a physiological coagonist of the NMDA subtype of glutamate receptor (47). The enzyme serine racemase has been shown to catalyze the conversion of L-serine to D-serine in rats and mouse Müller glia and in cortical astrocytes (46, 48). Furthermore, D-serine has been shown to play an important regulatory role in NMDAR response to light-evoked activity in retinal neurons (49). D-serine and serine racemase are mainly localized in Müller glia and retinal astrocytes (48), and in this regard, glia dysregulation of D-serine metabolism has been associated with retinal neurodegeneration, including glaucoma (47, 50).

### Glycolysis Is the Energetic Support of Müller Glia

Bioenergetic support is central to retinal homeostasis, as the retina is one of the most metabolically active tissues in the body (51). Although retinal neurons are highly dependent on mitochondrial phosphorylation to produce adenosine triphosphate (ATP), aerobic glycolysis has been shown to be the major provider of ATP in Müller glia (52). It is still unclear why glucose is not completely oxidized under aerobic glycolysis conditions in Müller glia. Some studies have suggested that the absence of the aspartate glutamate carrier (AGC) in Müller glia may explain the predominance of glycolysis in these cells (53, 54). Thus, AGC is a major component of the malate-aspartate shuttle (MAS) that translocates electrons produced during glycolysis to mitochondria to oxidize glucose (54). Despite some studies claiming that oxidative phosphorylation is less likely in Müller glia, we and others have observed that Müller glia switch primarily to mitochondrial respiration under glucose-deprived conditions (26, 55–58), whereas in the presence of sufficient intracellular glucose levels, Müller glia mainly rely on a combination of aerobic and anaerobic glycolysis (59, 60).

### Lactate Can Act as a Primary Energy Source

The predominant glycolysis during normal conditions results in the aerobic conversion of glucose into lactate, which is thought to be shuttled to the RGCs. The shuttling of lactate from glia to retinal neurons was originally hypothesized by Pellerin et al. (61). Their hypothesis predicted that uptake of glutamate would trigger glycolytic production of lactate, which in turn would be released and taken up by the surrounding neurons to fuel oxidative metabolism (62). It is clear that the lactate shuttle alone cannot explain the complex partnership between Müller glia and RGCs. Although lactate is highly produced in Müller glia, the overall role of lactate is likely to be greater in the retina compared with other tissues. Hence, the content of lactate is much higher in the retina compared with other tissues (16), and studies have reported a preference for lactate as a primary energy source in both Müller glia and RGCs compared with glucose (58, 63, 64).

It is clear that lactate is at the crossroads between glycolytic and oxidative energy metabolism and that more studies are necessary to understand further the roles of lactate in retinal homeostasis and pathology (65).

### Mitochondrial Dysfunction Is Associated With Glaucoma

Nevertheless, lactate metabolism is tightly associated with mitochondrial function, and disturbances in such have been identified as important to numerous retinal and optic nerve diseases, including glaucoma (52, 66, 67). Thus, age-related impairments of mitochondrial function may exacerbate these diseases (67–70). Mitochondrial genetic variations have been associated with primary open-angle glaucoma (POAG) in large genetic studies (71). In particular, genes involved in mitochondrial lipid and carbohydrate metabolism pathways have been implicated in the pathogenesis of POAG and normal-tension glaucoma (72). In line with these findings, metabolomic studies have also identified dysfunctional carbohydrate metabolism in POAG (73). In parallel, Müller glia seems to be vulnerable to the effects of metabolic stress and mitochondrial dysfunction (52, 54, 59, 74, 75). Because many of the essential functions of Müller glia are energy-dependent, mitochondrial dysfunction leaves these cells vulnerable during glucose restriction (52, 59).

### Retinal Diseases Cause Müller Gliosis and Nitric Oxide Production

As Müller glia span the entire depth of the neural retina, they are vulnerable to most forms of retinal injury. Accordingly, Müller gliosis, characterized by increased expression of the glial fibrillary acidic protein and activation of extracellular signal-regulated kinases, occurs in a wide range of retinal diseases (23, 76–78). Immediately after injury, Müller gliosis may be neuroprotective due to the production and release of antioxidants and trophic factors, including expression of ciliary body-derived neurotrophic factor (CNTF) (79). In contrast, later-stage gliosis has been associated with cell death and the establishment of a glial scar that inhibits neuronal regeneration (15, 80, 81). A particular pathological event during gliosis is the accumulation of nitric oxide (NO). Thus, NO has been shown to cause intracellular damage by inhibiting mitochondrial function, lowering ATP, and *via* direct damage to DNA. Furthermore, in a rat experimental glaucoma model, NO synthase (NOS) levels were found to be increased at the optic nerve head in response to IOP increase, resulting in increased NO levels (82). Despite the substantial literature on the neurotoxic effects of NO, NO also plays an important role in regulating retinal vascular tone to match neuronal activity (83). Thus, although NO contributes to neurovascular coupling and retinal homeostasis, it may become injurious in excess and in the context of retinal injury.

Overall, there is a growing body of evidence indicating that Müller glia is essential for RGC survival and that Müller glial dysfunction and stress are important factors in the pathogenesis of glaucoma (26, 80).

## Astrocytes and Their Role in Retinal Ganglion Cell Homeostasis and Glaucoma

Retinal astrocytes, also called astroglia, link neurons to blood vessels and are located almost exclusively in the retinal nerve fiber layer (84). They have been found to provide structural and physiological support to optic nerve head axons (85) and modulate remodeling of the extracellular matrix in response to IOP elevation (86, 87). During retinal injury or in response to elevated IOP, astrocytes are activated, followed by morphological changes, such as cell body hypertrophy and loss of thick processes (17, 87). In addition, astrocyte processes have been shown to lose their parallel orientation and distribution once axons are lost (88).

### Astrocytes Have Several and Different Functions in the Retina

Retinal astrocytes supply bioenergetic substrates to RGCs *via* the glutamate/glutamine cycle and *via* the transport of lactate and pyruvate (88, 89). It is thought that astrocytes account for more than 70% of the mitochondria in the optic nerve head (90, 91). In this context, astrocytes have been shown to engulf and degrade dysfunctional axonal mitochondria, a process known as transmitophagy (92, 93). Moreover, astrocytes have been shown to remove ions and recycle neurotransmitters from the extracellular space (28). During various pathological events, including elevated IOP or simply during aging, astrocytes undergo gliosis, a process of neurochemical and morphological remodeling (90, 94). Astroglial activation in glaucoma has been shown to increase the expression of many factors, including endothelin-1 (ET-1), tumor necrosis factor-α (TNF-α), oxidative stress molecules, and trophic factors, e.g., CNTF, with varied neuroprotective and harmful properties (17, 25, 32, 95, 96).

### Activated Astrocytes Play a Role in Glaucoma

In the course of progressive glaucoma, reactive gliosis and inflammation may potentially promote regeneration and remodeling in the optic nerve (90, 94). Within this era, IOP-induced mechanical stress has been shown to upregulate epidermal growth factor receptor after activation of astrocytes and finally leading to a neurodegenerative response with the upregulation of TNF-α, matrix metalloproteinases (MMPs), and endothelin and nitric oxide synthase-2 (NOS-2) (97–100). Upregulated expression of the phagocytosis-related gene Mac-2 (101) and ET-1 have also been described in experimental glaucoma as well as in the plasma and aqueous humor of glaucoma patients, indicating an association between these molecules and phagocytic degeneration of myelin in the optic nerve head transition zone in glaucoma patients (14, 92).

### Astrocytes Are Involved in Retinal Homeostasis

Overall, there is considerable evidence that astrocytes are essential for the maintenance of retinal homeostasis by clearing debris from RGC axons, supporting RGCs with energy substrates, and finally by removing excess neurotransmitters from the synaptic cleft (89, 95, 102). In contrast, activated astrocytes may have detrimental properties such as secretion of neurotoxic molecules and induction of inflammatory responses (14, 17, 95, 103, 104). Despite the lower abundance of astrocytes compared with Müller glia in the retina, the two macroglia subtypes have multiple overlapping functions but also separate properties that, in most cases, remain unknown. Future studies are highly needed to investigate the individual and the similar functions of both astrocytes and Müller glia. Moreover, their potential partnership is also important to understand. Current knowledge of such a partnership is discussed later.

## Oligodendrocyte and Their Essential Support of Retinal Ganglion Cell Axons

Oligodendrocytes myelinate axons in the CNS (31), significantly reducing the energy requirements for the propagation of action potential and further protecting against various cytotoxic and excitotoxic factors (105, 106). A single oligodendrocyte can produce numerous myelin segments on multiple axons (107). Myelinating oligodendrocytes express neurotransmitter receptors, ion channels, transporters, and gap junctions (108). RGC axons remain unmyelinated until they reach the retrolaminar portion of the optic nerve. At this point, the axons are ensheathed by and supported by oligodendrocytes (31).

### Oligodendrocytes Support Metabolic Transport

Oligodendrocytes are great examples of metabolic coupling between glia, which furnishes essential metabolic substrates to RGC axons (108, 109). Thus, oligodendrocytes have been shown to shuttle lactate to axons, thereby promoting axonal function and survival (107, 110). Both lactate and pyruvate are transported *via* monocarboxylate transporters (MCTs). MCT-1 transports lactate out of the oligodendrocyte membrane, whereas MCT-2 transporters are located on the RGC-axons and transport lactate into the RGC-axon (111). Astrocytes are also involved in lactate transport, as glucose is taken up in astrocyte processes from the blood vessels and metabolized to lactate/pyruvate, which is then transported to the oligodendroctyes via gap junctions and subsequently to the axons (107).

### Poor Myelination Is Involved in Neurodegeneration

An experimental link to the potential importance of impaired bioenergetic supply to axons in glaucoma has been provided. Thus, Rindholm et al. performed a study on mutant and transgenic mice with deficient proteolipid protein, a principal component of myelin, and showed that these mice had axonal degeneration, whereas the action potential propagation remained intact. Rindholm et al. further investigated the impact of myelin basic protein deficiency and reported that these mice lacked both compacted myelin and action potential propagation in the absence of axonal degeneration (112–114). Overall, these studies indicated that oligodendrocytes provide axons with support for survival and action potential propagation and that a dysbalance in oligodendrocyte myelination can be detrimental for RGC axon function and survival (112).

### Mitochondrial Dysfunction Is Associated With Glaucoma

Mitochondrial dysfunction has also been shown to play crucial roles in the homeostasis of oligodendrocytes. Thus, mitochondria are essential for the development of myelin sheaths and the development of carbon skeletons and lipid metabolism (115). Oxygen starvation and glucose deprivation have furthermore been shown to inhibit myelin development, and added together, multiple stressors have been found to have a detrimental impact on oligodendrocytes and thus associated with retinal neurodegeneration, such as seen in glaucoma (116).

In humans, optic nerve oligodendrocytes from glaucoma patients have been found to have smaller mitochondria compared with age-matched controls (116), supporting findings in the DBA/2J mouse model of glaucoma in which bioenergetic impairment was associated with axonal degeneration (114). Overall, there is some evidence for the involvement of oligodendrocytes in the pathogenesis of axonal dysfunction and loss. However, future studies are needed to investigate further how these glial cells predispose to axonal metabolic compromise and loss in glaucoma (117).

## Microglia and Their Impact on Retinal Ganglion Cell Survival and Function

Microglia are innate immune cells of the CNS. Activation of microglia may be triggered by multiple events (20), such as ATP release from nerve terminals, activated immune cells or damaged cells (118), neurotransmitter accumulation (119), the release of growth factors or cytokines (120), and changes in ion homeostasis (121). Activation of microglia is highly regulated. Hence, beneficial activation of microglia leads to the secretion of anti-inflammatory cytokines, such as interleukin (IL)-10 vs interleukin-10 (IL-10) that inhibits the production of pro-inflammatory cytokines by microglia (122–124). In contrast, marked activation of microglia in the setting of major insults results in the release of pro-inflammatory cytokines and cytotoxic agents, such as TNF-α, IL-1β, IL-6, inducible NOS, and NO, which in turn kill potential pathogens (125, 126) (Table 1).

**Table 1:** *Effector molecules of activated microglia*.

**Growth factors**	**References**
Basic fibroblast growth factor	(127, 128)
Transforming growth factor α	(129, 130)
Transforming growth factor β	(129)
**Cytokines**	
Interleukin-1α	(129, 131)
Interleukin-1β	(132, 133)
Interleukin-3	(130)
Interleukin-6	(134, 135)
Interleukin-10	(136, 137)
Tumor necrosis factor α	(132, 138)
**Complement factors**	
C1, C2, C4	(129, 139)
**Free radicals**	
Superoxide anions	(138, 140)
Nitrix oxide	(141, 142)

*Adapted from Minghetti et al. (143)*.

Activated microglia migrate toward sites of injury due to the expression of β-integrin CD11α (126) in a process mediated by the transcription factor nuclear factor-kappa-light-chain-enhancer of activated B cells (2). In addition, microglia support myelination, oligodendrogenesis, and neurogenesis, as well as stimulate synaptic formation and maturation (144).

### Activated Microglia and Glaucoma

Substantial evidence has shown microglial involvement in several eye diseases, including glaucoma (34, 145–148). In glaucoma, activated microglia have been described in clusters around blood vessels in the injured optic nerve and choriocapillaris, indicating an innate immune activation (149, 150). Dual roles for activated microglia have been proposed in glaucoma. On the one hand, activated microglia can phagocytose degenerated or dying RGCs, thereby maintaining a retinal environment free of toxic molecules (33), as well as the ability to secrete neurotrophic factors, such as brain-derived neurotrophic factor (BDNF) and CNTF (151), which provide neuroprotection and possibly promote neuroregeneration (95). On the other hand, chronic microglial activation leads to the release of neurotoxic and pro-inflammatory molecules, as mentioned earlier (28). In rodent glaucoma models, where mice were exposed to either hypoxic damage or ocular hypertension, a release of TNF-α and IL-1β from activated microglia accompanied by apoptosis of RGCs was found, supporting the involvement of microglia in glaucomatous neurodegeneration (152, 153). In addition, in an experimental rat model of glaucoma, microglial activation was shown to increase inducible NOS expression, NO production, and RGC injury (154). Activated microglia have furthermore been shown to release reactive oxygen species and prostaglandin E_2_, which predispose to RGC apoptosis (155). In glaucoma, microglial cell activation at the optic nerve head has been shown to be associated with altered cellular morphology, protein expression, and antigen-presentation (149, 156). Moreover, damage-associated molecular patterns (DAMPs), released by RGCs or by astroglia in the optic nerve head, which can trigger an inflammatory response, have been shown in response to elevated IOP (32). Finally, microglial activation has been correlated with axonal degeneration in an experimental glaucoma model (33).

### Microglia, Friend, or Foe?

As with macroglia, significant research has suggested that microglia can be considered either friend and foe depending on the degree of activation and context (21, 77, 143, 157, 158). One mechanism by which microglial activation is controlled is *via* a series of cell surface receptors (33). Thus, microglia are usually activated only when necessary to minimize safety damage to neighboring cells (159). Consequently, damage to RGCs may occur as observed in glaucoma when microglial homeostasis is disrupted. The expression of several inhibitory receptors decreases with age. Examples of such receptors are CX3CR1 and CD200. Ligand binding to CX3CR1 is crucial for the elimination of damaged or dying cells, whereas ligand binding to CD200 receptors leads to modulation of activated microglia during chronic as well as acute inflammation (138, 159). Other receptors vary with sex and age, such as the purinergic receptors P2 that bind to ATP to mediate intercellular communication (159). In a mice model of glaucoma, it has been demonstrated that a deficient activation of CX3CR1 enhances microglia activation and leads to neurotoxic loss of RGCs (34).

Emerging evidence exists on the impact of autophagy in microglia activation (160). In this context, autophagy modulation is thought to regulate microglia phagocytosis and inflammatory response (160, 161). Autophagy can either be considered as pro-inflammatory or anti-inflammatory depending on the acute or chronic stage of the injury (161). Potentially, the balance between autophagy and microglia can be regulated by pharmacological inhibition of, e.g., 3-methyladenine. Finally, studies indicate that some humans are genetically impaired of basal autophagy, which can impact retinal homeostasis and potentially promote retinal neurodegeneration, hereunder glaucoma (162–164). Future studies are needed to investigate further the partnership between autophagy and microglia as well as to elucidate further the interaction between microglia as well as other glia subtypes and RGCs.

## Retinal Glial Interactions

**Figure 3:**
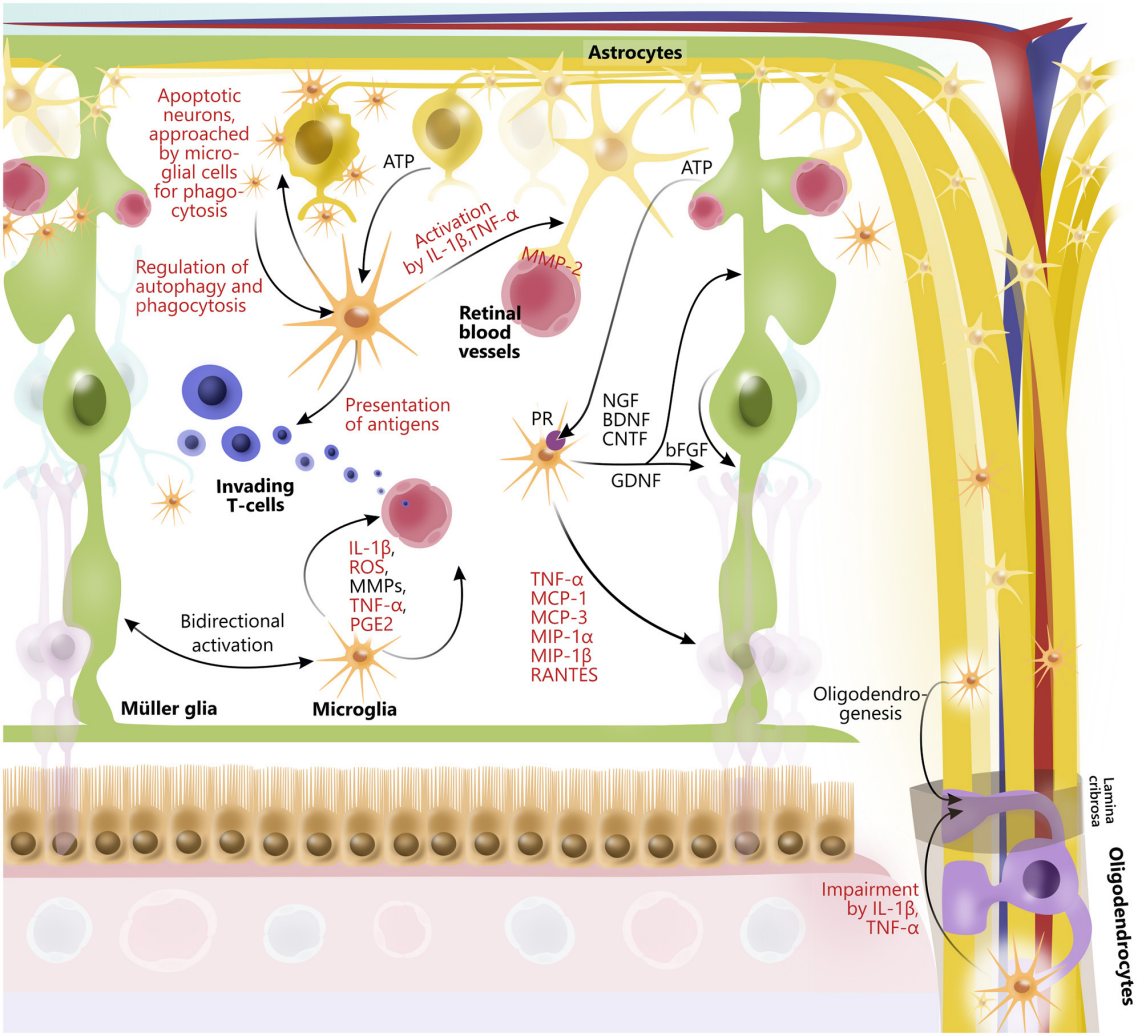
Glial interactions in retina and optic nerve. In retina, astrocytes, Müller glia, oligodendrocytes, and microglia widely interact to maintain retinal homeostasis by release of trophic factors and cytokines, ATP-exchange, phagocytosis of neuronal debris, antigen presentation, and by promoting activity of each other. Furthermore, retinal glia cells are interactively involved in maintaining retinal vessels as well as blood–retinal barrier, for example, through interactions between microglia and astrocytes. Some glial interactions also impair function of other glial cells. In particular, microglia *via* release of cytokines (IL-1β and TNF-alpha) affect function of oligodendrocytes, which myelinate axons of retinal ganglion cells in optic nerve. However, microglia can also contribute to oligodendrogenesis. Although glial interactions attempt to maintain retinal homeostasis, they can also promote retinal neurodegeneration. Prodegenerative factors released from glial cells to interact with one another are highlighted in red.

Emerging evidence has identified the importance of cross talk between retinal glial cells in health and disease (14, 165–167) (Figure 3). In general, glia interactions attempt to maintain retinal homeostasis and regulate each other's activity. However, glia interactions can also create imbalance and thus contribute to retinal neurodegeneration.

An example of a glia interaction is the modulation of T cell response due to microglia antigen presentation (165), which in turn regulate the inflammatory cytokine levels, including TNF-α and IL-1β, followed by astrocyte activation (28, 168). Furthermore, microglial migration and immune cell recruitment have been correlated with Müller glial activation (14), indicating tight coordination of retinal immune responses (169). In line with this, activated microglia secrete TNF-α, CCL2 (MCP-1), MCP-3, MIP-1α, MIP-1β, and CCL5 (RANTES), which mediate activation and recruitment of additional microglia, amplifying the inflammatory response (84, 165, 170). In addition, microglia-derived IL-1β has been shown to upregulate the expression of Ccl2, Cxcl1, and Cxcl10 in Müller glia, which has been associated with retinal neurodegeneration (171). Another example of microglia and Müller glia interaction is the secretion of microglia-derived nerve growth factor (NGF), BDNF, and CNTF from microglia that in themselves protect RGCs (172) but also modulate the production of basic fibroblast growth factor and GDNF in Müller glia, conferring neuroprotection (167, 173). Both astrocytes, microglia, oligodendrocytes, and neurons secrete MMPs (174). Under normal conditions, astrocytes and microglia express MMP-2 (gelatinase A) in the foot processes near blood vessels (175). Upon astrocyte and microglial activation, MMP-2 is, however, increased, thereby causing increased permeability of the blood–retinal barrier (176), angiogenesis, and glial scar formation (177). Activated microglia also express MMP-3 (stromelysin-1), which in turn activates proMMP-9 (178). MMP-9 has been found to be elevated when there is an increase in the blood–retinal barrier permeability. In line with this, an MMP-9 increase has been shown in diabetic rat retinas when glucose levels rise (176). MMP-9 has also been implicated in myelin basic protein degradation, and it has therefore been suggested that MMP-9 is associated with demyelination and axonal injury (178–180). Finally, both the effects of MMP-9 and MMP-3 have been shown to be enhanced by TNF-α and IL-1, further indicating complex interactions between molecules secreted by different retinal glia (181–183).

Glia–glia interactions have also been shown between oligodendrocytes and microglia as well as astrocytes. In this context, studies have shown that microglial activation can cause astrogliosis and apoptosis of oligodendrocytes *via* TNF-α and IL-1β, causing an increase in MMP production in astrocytes and microglia as well as a possible induction of oxidative stress, inflammation, and glutamate toxicity (183–185).

Although there is currently relatively sparse literature on glia–glia interactions, it can be expected that this interaction will prove to be very relevant for both the understanding of retinal homeostasis and for neurodegenerative diseases such as glaucoma (186).

## Therapeutic Approaches

### Targeting Retinal and Optic Nerve Glial Cells to Treat Glaucoma

Glial cells in the retina and the optic nerve play an important role in supporting RGCs and their axons, and because glial cell dysfunction has been implicated in glaucoma, it is conceivable that future treatments for glaucoma may target glial cells.

### Targeting Müller Glia and Astrocytes

#### Modulation of Glutamate via N-Methyl-D-Aspartate Receptors

RGC damage leads to elevated glutamate levels, causing NMDARs to be overstimulated with a consequent increase in Ca^2+^ influx and excitotoxicity (187). Impaired clearance of glutamate by Müller glia is involved in the pathogenesis of both glaucoma and diabetic retinopathy (37, 188, 189). Amidation and oxidation are the two routes of glutamate disposal. In a cultured rat retinal Müller cell line, treatment with hydrocortisone was shown to increase the amidation of glutamate to glutamine, whereas the addition of branched-chain keto acids was found to enhance oxidation of glutamate, suggesting that intracellular levels of glutamate play a role in the removal of extracellular glutamate (35). In a rodent model of ocular hypertension, the use of memantine nanoparticles, a non-competitive NMDAR antagonist, conferred RGCs neuroprotection (190). Unfortunately, oral memantine did not show any significant prevention of glaucoma progression when tested in a clinical trial (191). Another modulator of glutamate-induced toxicity, brimonidine, has been proposed to slow the rate of glaucoma progression in glaucoma patients in an additive manner other than its IOP-lowering effect (192). In a rodent model, brimonidine was shown to modulate glutamate uptake by glial cells after induction of ocular hypertension, suggesting neuroprotection through modulation of macroglia (193).

#### Neurotrophin Administration

Neurotrophins are important in the development, differentiation, and survival of RGCs. Many of these neurotrophins are produced by glial cells during normal conditions. In this context, BDNF has been shown to protect RGCs in mice with ocular hypertension (194). Furthermore, it has been shown that GDNF combined with BDNF convey synergic protective effects (195). Finally, glial cells are important producers of neurotrophins, including NGF, BDNF, CNTF, neurotrophin-3, and neurotrophin-4/5, all having potential as neuroprotective properties (196). Among the mentioned neurotrophins, more have already been tested in preclinical settings. An example is mature BDNF, which has been targeted to secretory vesicles within RGCs by adeno-associated virus gene therapy, increasing BDNF production and long-term BDNF receptor expression in a mouse model of optic nerve damage and in a rat model of chronic IOP, which provides neuroprotection against RGCs (197). In addition, increased release of CNTF by Müller glia has been shown to provide endogenous neuroprotection of RGCs after both ischemias and in response to the induced ocular hypertension (194). Recently, an encapsulated cell technology has allowed a controlled, continuous, and prolonged administration of CNTF in animal models that provide photoreceptor protection (198). In general, the administration of exogenous neurotrophins or the augmentation of endogenous production has been shown to have a protective effect on RGCs in several experimental models, highlighting this as a potential therapeutic strategy for glaucoma (199). The efficacy of such treatments may, however, decrease over time as treatment with chronic neurotrophin administration can lead to downregulation of the relevant receptors (197).

#### Targeting Astrocytes and Astrocyte Activation

Astrocyte activation may be an important factor in the pathogenesis of glaucoma. In this context, inhibition of astrocyte activation has been shown to increase neuronal survival in experimental glaucoma models *via* modulation of a tyrosine kinase inhibitor of epidermal growth factor receptor (200) or blocking of endothelin-1 (201). Similarly, the neuroprotective effects of calcium channel blockers and endothelin blockers in humans with glaucoma are thought to act *via* this mechanism (202).

In addition to the previously mentioned involvement of TNF-α in microglia activation, TNF-α has also been shown to mediate both astrocyte, Müller glia, and oligodendrocyte activation followed by RGC death (203–205). In a rodent glaucoma model, this detrimental effect of TNF-α was demonstrated after intravitreal TNF-α injections and reversed by an antibody neutralizing TNF-α activity or by deleting the genes encoding TNF-α or its receptor, TNF-R2 (204). Future studies are needed to define further the potential role of TNF-α inhibitors as a treatment target for neuroprotection (206).

Nutrition may affect retinal homeostasis and, in particular, mitochondrial function. Hence, a ketogenic diet was shown to increase mitochondrial respiration, hereunder mitochondrial respiration in astrocytes (207, 208). In addition, the ketogenic diet has been reported to restore monocarboxylate transporters to boost the antioxidant response followed by preservation of RGC function and structure without affecting glycogen stores (117). Finally, a low-carbohydrate diet has been shown to reduce the risk of POAG in a US cohort (209). In summary, nutrition and ketogenic diets may increase the resistance toward glaucomatous neurodegeneration. Future studies are, however, necessary to further investigate such potential.

### Targeting Oligodendrocytes

#### Inhibition of Inflammatory Mediators

Oligodendrocyte degeneration has been correlated to TNF-α release followed by increased IOP and optic nerve damage. In this context, suppression of TNF-α, with an anti-TNF-α blocking antibody or the deletion of the gene encoding TNF-α, was shown to elicit neuroprotective effects in the optic nerve and RGCs in a mouse model of glaucoma (204).

#### Inhibiting Lingo-1

LINGO-1 is a leucine-rich repeat, expressed on oligodendrocytes and neurons. It negatively regulates differentiation and myelination, neuronal survival, and axonal regeneration. In glaucoma models and human CNS diseases, LINGO-1 expression has been found to be upregulated (210). LINGO-1 has furthermore been shown to have negative regulatory functions in axonal regeneration, neuronal survival, and oligodendrocyte differentiation as well as myelination. In addition, LINGO-1 was found to be increased in models of spinal cord injury and glaucoma (211). The LINGO-1 monoclonal antibody, BIIB033, has shown promise as a neuroprotective and neuroregenerative strategy in clinical studies, but continued evaluations are needed to confirm this promising effect in glaucoma patients (211).

### Targeting Microglia

#### Inhibition of Microglial Activation

Microglia have increasingly been identified as targets in glaucoma neuroprotection and neuroregeneration (22, 28, 212, 213). The blockade of the microglial adenosine A_2A_ receptor has been shown to protect RGCs from elevated IOP in murine glaucoma models by controlling the microglial activation and inhibiting reactive oxygen species (214). Minocycline has also been shown to inhibit microglial activation and upregulate pro-survival genes in experimental glaucoma (215, 216). Although minocycline has proven oral safety and has been found to cross the blood–brain barrier, long-term randomized control trials with the necessary high doses of minocycline are needed (217).

#### Modulation of Microglial Activation

In addition to the harmful effects of microglia, the activation of these cells can also benefit the healthy eye, where such activation, e.g., acute inflammation, has been shown to protect against neuronal damage (22). Microglia activation is regulated by several inhibitory pathways, such as the ligand fractalkine (FXN or Cx3cl1) in neurons and the receptors Cx3cr1 and CD200R in microglia (218). The loss of Cx3xl1 signaling has been shown to exacerbate dysfunctional axon transport in RGCs, increased CCR2+ macrophages infiltration (219), and upregulation of NOS-2 in myeloid cells from DBA/2J mice (219).

CD200 is expressed in the vascular endothelium of the retina, photoreceptors, and RGCs (34) and interacts with CD200R to modulate microglial activation (220). Breakdown of CD200-CD200R is involved in RGC loss in experimental glaucoma (221). Thus, in a rat model with optic nerve crush, the agonist of CD200R known as CD200Fc was shown to increase CD200R expression and inhibit CD200 expression, thereby assisting in the neuroprotection of RGCs (222, 223). Overall, microglial activation may be beneficial for RGC function and survival, and numerous studies emphasize the dual roles of microglia activation. However, more studies are needed to further understand such potential beneficial functions of microglia in glaucoma.

### Inhibition of Inflammatory Mediators

In advanced glaucoma, inflammatory mediators, including TNF-α, are increased. Inhibition or genetic deletion of TNF-α reduces the activation of microglia (32, 224), and the blockade of TNF-α signaling has been shown to protect RGCs in an experimental glaucoma mouse model (204, 225).

The Fas ligand (FasL), a member of the TNF protein family, links microglia activation and the induction of apoptosis of RGCs through the Fas receptor. In the eye, FasL can be expressed as the membrane-bound pro-apoptotic and pro-inflammatory protein (mFasL) or as the soluble, non-apoptotic, and non-inflammatory form sFasL (226). Previous studies have shown that FasL is constitutively expressed in ocular tissue, where the ligand helps maintain the immune-privileged state of the eye and helps prevent neoangiogenesis. However, FasL has also been shown to play an important role in retinal neurotoxicity. In this context, FasL has been shown to accelerate RGC death in an experimental glaucoma model (227). In line with this, the peptide inhibitor of the Fas receptor, ONL1204, has been found to halt mFasL activation by inhibition of microglial activation, inflammation, and apoptosis of RGCs in a mice model (228). In contrast to the neurotoxic effects of FasL, FasL administration has been shown to protect RGCs from cell death (227). Overall, the contradictive roles of FasL and mFasL require more studies to investigate the potential roles of FasL/mFasL modulation in future neuroprotective treatments.

#### Reducing Oxidative Stress

Orally administered docosahexaenoic acid and intravitreal injection of polysialic acid has been shown to reduce microglial activation by decreasing oxidative stress and inflammation in rodent models of glaucoma and experimental glaucoma models (146). Moreover, the natural resin mixture, propolis, produced by honeybees, has been shown to reduce neuroinflammatory responses and reduce oxidative stress in microglia cell cultures by inhibiting nuclear factor-κB when cultures were exposed to hypoxia (229).

#### Macrophage Pro-Inflammatory Cytokines

Glial cell activation triggers macrophage infiltration and the release of pro-inflammatory cytokines that further activate glial cells. Such pro-inflammatory cytokines have been found to be upregulated in both the blood and in the aqueous humor of patients with POAG (133, 230). Accordingly, treatments targeting macrophage-derived pro-inflammatory cytokines, such as IL-1β, may be used in the future treatment of glaucoma (133, 194).

## Conclusion

Glial cells play complex and multifactorial roles in glaucoma. Advances in our understanding of the nature and regulation of these various roles in health and disease have enabled the identification of novel therapeutic strategies to protect RGCs in glaucoma. Therapies targeting glial cells or those emulating the protective effects of these cells on RGCs will likely go hand-in-hand with conventional therapies to lower IOP and with emerging approaches that aim to augment neuronal bioenergetic resilience and promote axonal repair.

## Author Contributions

MG-B has search the literature and written the first draft. MK has been main responsible for the process. ZM has designed the figures. KM, PW, and KF have all been participating in critical commenting and proof reading. All authors have contributed to the writing process.

## Conflict of Interest

The authors declare that the research was conducted in the absence of any commercial or financial relationships that could be construed as a potential conflict of interest.
